# Antioxidative responses during germination in quinoa grown in vitamin B-rich medium

**DOI:** 10.1002/fsn3.211

**Published:** 2015-03-09

**Authors:** Andrea Pitzschke, Anna Fraundorfer, Michael Guggemos, Norbert Fuchs

**Affiliations:** 1 Division Plant Physiology, Department Cell Biology, University of SalzburgSalzburg, Austria; 2 Department Applied Genetics and Cell Biology, University of Natural Resources and Life Sciences (BOKU)Vienna, Austria; 3 Institut für NährstofftherapieUnternberg, Austria

**Keywords:** Antioxidants, nutritional value, quinoa, vitamins

## Abstract

Synthetic vitamin preparations have grown in popularity to combat health risks associated with an imbalanced diet, poor exercise and stress. In terms of bioavailability and diversity, they lack behind vitamins naturally occurring in plants. Solutions to obtain plant-derived vitamins at a larger scale are highly desirable. B vitamins act as precursors of enzymatic cofactors, thereby regulating important metabolic processes both in animals and plants. Because during plant germination, the vitamin content and micronutrient availability increase, sprouts are generally considered a healthier food as compared to dry grains. Germination only occurs if a plant′s antioxidant machinery is sufficiently activated to cope with oxidative stress. Seeds of quinoa, an edible gluten-free plant naturally rich in minerals, germinate readily in a solution containing the eight B vitamins. We studied biochemical changes during quinoa germination, with a focus on nutritionally relevant characteristics. The results are considered from a nutritional and plant physiological perspective. Germination of quinoa in vitamin-rich medium is a promising strategy to enhance the nutritional value of this matrix. Additional health-beneficial effects indirectly resulting from the vitamin treatment include elevated levels of the multi-functional amino acid proline and a higher antioxidant capacity. Plant biomolecules can be better protected from oxidative damage in vivo.

## Introduction

A sedentary life style, imbalanced diet and excessive stress negatively affect human health. The general awareness of this problem is growing; and many consumers aim to compensate for their unhealthy life style through oral intake of synthetic vitamin preparations. However, synthetic vitamins have an overall lower bioactivity and availability, and they can certainly not fully replace naturally healthy plant-derived food. Furthermore, several clinical symptoms have been associated with overdose of particular vitamins (Chawla and Kvarnberg 2014). In plants, the individual vitamins hardly occur in a single, pure form, but chemically modified or as part of various organic complexes. This natural vitamer diversity reflects the high nutritional value of certain plant food. Vitamin production in transgenic plants is principally feasible (Herbers 2003), but clearly lacks general acceptance. Alternative solutions to obtain plant-specific vitamin derivatives at a larger scale are therefore desirable.

Vitamin deficiency, overdose or imbalances are an issue for plants, too (Smith et al. 2007). Flexible adjustments of vitamin biosynthesis and metabolization partially account for the ability of plants to respond and adapt to environmentally challenging situations (Asensi-Fabado and Munne-Bosch 2010). One major beneficial characteristic of several plant-derived vitamins is their ability to act as antioxidants (reviewed recently by Asensi-Fabado and Munne-Bosch 2010). Phenolic compounds such as *α*-tocopherol (vitamin E) and flavonoids are well known for their antioxidative effects (Pandey and Rizvi 2009). The multifunctional amino acid proline deserves particular attention in redox biology, plant physiology and food research (Szabados and Savoure 2010; Ben Rejeb et al. 2012).

B vitamins are a group of water-soluble vitamins comprising thiamin (B1), riboflavin (B2,) niacin or niacinamide (B3), panthothenic acid (B5), pyridoxin or pyridoxal (B6), biotin (B7), folic acid (B9) and cobalamin (B12). B vitamins work collectively and individually in every cell; they are primarily important for cell metabolism. Supplements containing all eight are referred to as vitamin B complex. Overdose of individual members of the B vitamin group, particularly vitamin B6 can have adverse effects (Lheureux et al. 2005; Chawla and Kvarnberg 2014). It is reasonable to assume that B vitamins are of optimal value for human nutrition if they do not exceed a certain threshold, and if a balanced ratio exists between the individual members. With the exception of vitamin B12, formed by bacteria only, B vitamins are naturally found in plants. Some foods are especially good sources of just one vitamin.

Similar to the situation in humans, several members of the vitamin B group act as precursors of enzymatic cofactors in plants, thereby regulating important metabolic processes. Plant mutants with impaired vitamin biosynthesis or a disturbed vitamin balance show severe growth deficits. For example, consistent with the vital function of vitamin B6 for the general well-being of plants, *Arabidopsis thaliana* mutants with dysfunctional vitamin B6 biosynthesis genes show aborted seeds that do not germinate (Titiz et al. 2006).

Reactive oxygen species (ROS), such as hydrogen peroxide (H_2_O_2_), superoxide anion (O_2_^−^) and hydroxyl radical (HO^.^) play key roles in signal transmission and regulate a plethora of cellular processes in animals (Rajendran et al. 2014) and plants (Pitzschke et al. 2006). However, in any living organism excessive amounts of ROS are destructive to biomolecules (DNA, proteins, lipids, polyphenols) and will ultimately cause cell death (Mittler 2002). Oxidative stress results from an increase in ROS production and/or a decrease in the cellular antioxidant capacity. In animals, the oxidative damage triggered by ROS has been implicated in the cause of numerous diseases and accelerated aging (reviewed in Lobo et al. 2010; Ray et al. 2012). Due to the dual role of ROS, production and removal needs to be tightly regulated. This is mediated through a sophisticated network of antioxidants and antioxidant enzymes. Plants with an elevated antioxidant capacity are generally more tolerant toward stress. Reciprocally, high stress resistance is associated with an efficient ROS removal system. Oxidation diminishes the nutritional value of any food. To supplement processed plants (postharvest) with antioxidants (e.g., ascorbic acid) is a means to prevent oxidative decay (e.g., “rancidity” and off-flavor) and to prolong shelf life. However, because ROS are constantly being produced while a plant is alive, oxidative damage that had occurred in vivo cannot be reverted through subsequent addition of antioxidant agents.

Adverse environmental conditions (e.g., temperature or osmotic stress) generally block seed germination and development. The degree to which seeds tolerate such “challenges” is species dependent; it is based on altered stress perception and transduction. In viable seeds, ROS are required for seed dormancy release (Bailly et al. 2008a). The mobilization of storage lipids by ß-oxidation and concurrent increase in ROS pose a particular oxidative challenge to the developing seedling. During seed germination, vitamin content and micronutrient availability increase substantially (reviewed in Bohn et al. 2008; Hotz and Gibson 2007). Seedlings (“sprouts” are therefore considered a healthier food as compared to dry grains. A fascinating question is whether the nutritional value can be increased further by adjusting the conditions during seed germination.

Seeds of Quinoa (*Chenopodium quinoa*) are an important food source in South American countries. Quinoa has attracted increasing attention by the European health food industry. Besides being gluten-free, quinoa has further health-beneficial properties (Vega-Galvez et al., 2010; Laus et al. 2012), making it attractive not only for celiac patients but for the general public. Quinoa germinates exceptionally fast (radicle protrusion within few hours), and it is able to grow in areas too dry or too saline for the major cereal crops. Quinoa seedlings have been reported to efficiently absorb exogenously applied minerals such as Li, Fe, Co, and Se. Quinoa can germinate even in the presence of high electrolyte concentrations (Lintschinger et al. 1997). This plant is therefore an important research object in agricultural, plant physiology and food science.

A patent exist on the treatment of germinating quinoa with a vitamin B complex solution. The resultant seedlings contain elevated amounts of B vitamins, which remain present after wash cycles (Fuchs et al. 2007), Table1. The physiological and metabolic responses to vitamin treatment have remained elusive till now. Presumably, these seedlings can maintain a certain vitamin balance by selectively regulating absorption, metabolization, sequestration and turn-over of individual vitamins. These adaptation responses undoubtedly would require active participation of several plant proteins and thus a certain cellular reprogramming.

**Table 1:** Vitamin concentrations in the nutrient solution used for seed imbibition, and in seedlings after excessive washing and drying. Data source: patent specification (Fuchs et al. 2007)

B vitamins	mg/L nutrient solution	mg/100 g seedling dry weight
Thiamine	1500	83.4
Riboflavin	10,000	134.0
Niacin	22,000	1300.0
Pantothenic acid	25,000	793.0
Pyridoxine	3300	155.0
Biotin	250	14.9
Folic acid	1000	12.4
Cobalamin	5	0.21

The present study investigates germination-related changes of quinoa at the molecular level. Seeds and seedlings germinated in either water or a vitamin B complex solution are compared with respect to their overall protein profile, superoxide levels, antioxidant enzyme activities, and proline content.

## Materials and Methods

Quinoa seeds (ecotype “Real”, grown in Bolivia) were obtained from Fairtrade, Austria. The procedure of vitamin treatment, washing and drying has been described in a previous patent specification (Fuchs et al. 2007). Briefly, seeds are imbibed in a vitamin B complex solution. Two-day-old seedlings are washed intensively to remove loosely adherent vitamins, and subsequently dried. Details on vitamin concentrations in the nutrient solution and in vitamin-enriched dried seedlings are shown in Table1. Water-germinated seedlings (Q_H_2_O), were produced in the same way, using tap water instead of vitamin B complex solution.

### Protein extraction

Quinoa seeds or dry seedling material were frozen in liquid nitrogen and ground to a fine powder using a tissue-lyser mill (Retsch). For each extraction, plant material was freshly ground and processed directly. The powder was thoroughly mixed with two volumes of prechilled extraction buffer (four buffers with different compositions tested, listed below). After 10 min incubation on ice, soluble and nonsoluble fractions were separated by centrifugation (4°C, 10,000 *g*, 15 min). Samples were kept on ice throughout. Protein content was assessed spectrometrically, using the Bradford reagent (Bradford 1976) and BSA as a standard. For protein profile analysis, samples were denatured by 5-min incubation at 95°C in loading dye (60 mmol/L Tris-Cl pH 6.8, 2% SDS, 10% glycerol, 5% *β*-mercaptoethanol, 0.01% bromophenol blue; added as fivefold concentrate). About 10–20 *μ*g protein samples were loaded onto 12% polyacrylamide gels and separated at 15 mA. Gels were subsequently stained with Coomassie blue, using the “microwave method” (Wu et al. 2007).

Extraction buffers for protein profiling and enzyme activity assays:

Buffer 1: 50 mmol/L Tris/HCl, pH 7.5, 5 mmol/L EDTA pH8, 5 mmol/L EGTA pH8, 2 mmol/L DTT, 100 mmol/L ß-glycerophosphate, 10 mmol/L NaVO_3_, 10 mmol/L NaF, 10 mmol/L PMSF, 10 *μ*g/mL aprotinin, 10 *μ*g/mL leupeptin, 2 mmol/L DTT
Buffer 2: 50 mmol/L sodium phosphate pH 7, 1 mmol/L EDTA, 1% PVPP, 1 mmol/L ascorbic acid (AsA)
Buffer 3: 50 mmol/L Tris pH 7.5, 20% glycerol, 1 mmol/L AsA, 1 mmol/L DTT
Buffer 4: 100 mmol/L Tris pH 8.5, 2 mmol/L CaCl_2_, 0.1% triton

### Enzyme activity assays

Fresh, ice-stored protein extract (concentration adjusted to 5 *μ*g/*μ*L) was supplemented with reaction buffer. Samples were briefly mixed and absorbance was measured immediately and at regular intervals using a TECAN m200-infinite instrument. Enzymatic reactions were incubated at room temperature. Control reactions contained the respective volume of extraction buffer. Different amounts of protein extract were tested to rule out possible inhibitive effects of extract components. Specific enzyme activities are expressed as unit/mg protein. One unit is 1 *μ*mol substrate conversion per minute. Data are shown as mean ± SD of three measurements that were conducted with independent protein extracts.

#### Ascorbate peroxidase

About 5–10 *μ*g protein extract was mixed with 600 *μ*L reaction buffer (25 mmol/L sodium phosphate pH7, 0.1 mmol/L EDTA, 0.5 mmol/L ascorbic acid, 0.1 mmol/L H_2_O_2_). Ascorbate conversion into dehydroascorbate was monitored in UV-transmissible cuvettes by measuring the decline in absorbance at 290 nm (AsA: *ɛ* = 2.88/mm/cm).

#### Catalase

About 5–10 *μ*g protein extract was mixed with 600 *μ*L reaction buffer (100 mmol/L sodium phosphate pH 7.4, 10 mmol/L H_2_O_2_). H_2_O_2_ decomposition was monitored in UV-transmissible cuvettes by measuring the decline in absorbance at 240 nm (H_2_O_2_ : *ɛ* = 0.039/mm/cm).

#### Glutathione reductase

About 5–10 *μ*g protein extract was mixed with 200 *μ*L reaction buffer (100 mmol/L sodium phosphate pH 7.4, 1 mmol/L GSSG, 0.1 mmol/L NADPH). NADPH conversion into NADP was monitored in transparent 96-well-plates by measuring the decline in absorbance at 340 nm (*ɛ* = 6.22/mm/cm/). For assessing NADPH-converting activities other than glutathione reductase, GSSG was omitted from the reactions, and oxidation of NADPH was recorded at 340 nm (*ɛ* = 6.22/mm/cm). The enzyme activity was calculated as *μ*mol NADPH oxidized per min/mg of proteins.

#### Superoxide dismutase

Protein extracts were prepared in 25 mmol/L HEPES pH 7.8, 2% PVPP, 0.2 mmol/L EDTA and quantified by Bradford assay. In-gel superoxide dismutase (SOD) activity assays were performed according to (Kuo et al. 2013) with minor modifications. About 20 *μ*g protein extracts, supplemented with SDS-free protein loading dye, were separated on 10% native polyacrylamide gels at 4°C, 12 mA for 2 h. The gels had been prerun for 2 h to remove TEMED and APS. Gels were rinsed in distilled water, incubated in NBT solution (2 mmol/L NBT in distilled water) in the dark at room temperature for 15 min and rinsed again. Gels were subsequently incubated for 15 min (RT, dark) in riboflavin solution (28 *μ*mol/L riboflavin, 28 mmol/L TEMED, 0.1 mol/L sodium phosphate pH 7.8), briefly rinsed in distilled water and illuminated with a white-light box for 30 min at RT, followed by photography. White SOD activity bands appeared in the blue background. These bands result from prevention of NBT reduction by superoxide to blue formazane precipitates. For photometric measurements of SOD activities, 2 *μ*g protein extracts were mixed with 200 *μ*L reaction buffer (25 mmol/L HEPES 7.8, 10 mmol/L Methionine, 75 *μ*mol/L NBT, 0.2 mmol/L EDTA, 50 *μ*mol/L riboflavin) in a 96-well transparent plastic plate. Absorbance readings (OD560 nm) were taken immediately and after 30 min illumination. One unit SOD was defined as the amount of enzyme that prevented 50% of NBT reaction (control reaction with protein extraction buffer only).

### Superoxide detection

Quinoa seeds or dry seedling material were ground to a fine powder using a tissue-lyser mill (Retsch). About 20 mg powder was supplemented with 0.5 mmol/L NBT in 20 mmol/L HEPES, pH 7.0. Samples were mixed thoroughly several times. Formazane precipitation was documented by photography. Consistent results were obtained from all four independent repeats of the experiment.

### Proline quantification

Proline is very soluble and can be readily extracted in ethanol. A previously reported ninhydrin-based method (Carillo and Gibbon 2011) was used, with slight modifications. Screw-cap tubes containing 30–50 mg freshly powdered plant material and 300 *μ*L pure ethanol were heated at 95°C for 10 min under vigorous shaking. Samples were briefly cooled, and sample volumes were adjusted to 1 mL by adding 70% ethanol. After a second incubation at 95°C for 20 min, extracts were separated by centrifugation (5 min, 14,000 *g*) About. 60 *μ*L supernatant fluid was mixed with 140 *μ*L ninhydrin solution (1% ninhydrin, 60% ethanol, 20% acetic acid). Control (60 *μ*L ethanol) and standard reactions (dilution series of proline in 60 *μ*L ethanol) were set up in parallel. Samples were incubated in plastic strips in a PCR machine at 95°C for 40 min, cooled and transferred to a 96-well plate. Absorbance was recorded at 520 nm. Proline levels were calculated as mg proline per 100 g dry weight. Data are shown as mean ± SD of three independent measurements of ninhydrin reactions that were set up with independently prepared extracts.

## Results

**Figure 1:**
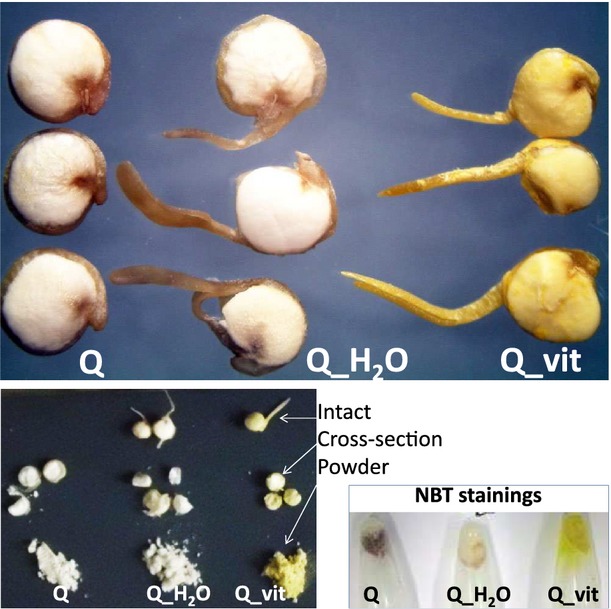
Top: Three representatives each of quinoa seeds and seedlings after germination in water or in a vitamin B complex solution are shown. The yellow color in vitamin-treated seedlings (surface, cross-sectional areas and powdered material) derives from absorbed riboflavin (vitamin B2), which remains after the washing and drying procedure. Bottom: Superoxide detection. Equal amounts of seeds and seedlings were ground to a fine powder and immediately mixed with NBT staining solution. The experiment was repeated four times with similar results.

Throughout the study, three sample types were compared:

Nontreated quinoa seeds (“Q”); and 2-day-old seedlings germinated in water (“Q_H_2_O”) or in a vitamin B complex solution (“Q_vit”) (Fig.1). Seedlings were washed thoroughly (in order to remove nonabsorbed vitamins) and subsequently dried. Dried seedling material was used, because this is the most convenient form for storage, transport and commercial use. The characterization of dried material is thus more relevant for the consumer. The three sample types were analyzed in parallel to distinguish general effects related to germination from vitamin treatment-specific responses. Owing to its yellow color, riboflavin can be observed directly. Cross-sectional areas of seedlings have the same yellow color intensity as the surface of intact seedlings (Fig.1, left). If the vitamin was only adsorbed to the seedling surface, that is, not taken up during germination, cross-sectional areas and powdered seedling material should have a whitish color (as in the corresponding samples from seeds and water-treated seedlings).

### Protein profile of seeds and seedlings

**Figure 2:**
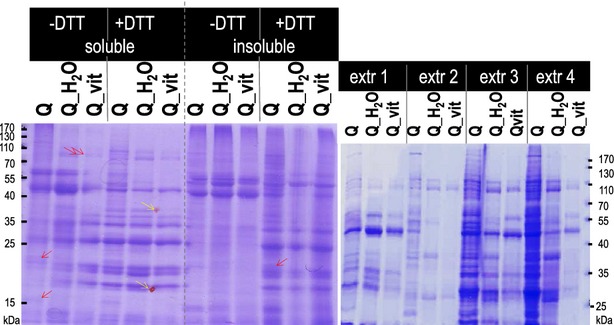
Protein profile of quinoa seeds and seedlings. Protein extracts were separated by SDS-PAGE. Left: Soluble and insoluble fractions (supernatant and pellet obtained after centrifugation) were denatured in loading dye. Extraction buffer and loading dye with and without the reducing agent DTT were used. Right: Protein extraction using four different buffers. Because the prime interest was in qualitative differences between samples, protein concentrations had not been adjusted here. Arrows indicate examples of germination-or vitamin treatment-specific bands.

Upon imbibition of viable seeds, seed-stored nutrients are mobilized to provide energy to the developing seedling. Seed storage proteins are degraded, and germination-related genes are activated, triggering the biosynthesis of the corresponding proteins (Catusse et al. 2008). To monitor the germination process of quinoa at the protein level, proteins were extracted from Q, Q_H_2_O, Q_vit, separated by SDS-PAGE and visualized by Coomassie Blue staining (Fig.2). It is known that the efficiency to which certain proteins (e.g., membrane-bound; cytosolic) can be isolated depends on the particular composition of the buffer used for extraction (e.g., pH, detergents). In order to detect preferably many differences between the three quinoa samples, several extraction protocols were employed. The protein profiles of the nonsoluble pellet fractions remaining after extraction and centrifugation were also examined. Analyses were carried out under nonreducing and reducing conditioned, that is, in the absence or presence of dithiothreitol (DTT).

Figure2 displays the size distribution of Q, Q_H_2_O, Q_vit proteins obtained *via* several extraction procedures (see methods). For each extraction methods, several protein bands with altered abundance in the three samples were observable. Some protein bands occurred in Q_H_2_O and Q_vit but not in seeds and may thus be related to germination in general. Protein bands exclusively accumulating in Q_vit were also detected. In summary, changes in protein composition occur during quinoa germination. In response to the vitamin treatment seedlings specifically adapt the biosynthesis and/or turnover of several proteins. Food products containing ground quinoa seeds plus vitamin supplements would thus be clearly distinguishable from seedlings obtained from the above-mentioned procedure.

### Reactive oxygen species: superoxide

Given the known role of vitamin B members in redox balancing, differences in redox-related properties would be a first indication that the exogenously added vitamins were not only absorbed by quinoa but incorporated into functional derivatives. This issue was first examined using Nitroblue tetrazolium (NBT) stainings. NBT specifically reacts with superoxide, a highly aggressive type of ROS. In the presence of superoxide anions, the pale-yellow NBT is reduced to a dark blue-colored formazane product. To assess superoxide levels in quinoa, equal amounts of dry, freshly powdered Q, Q_H_2_O, Q_vit material were incubated in NBT staining solution (Fig.1 bottom). The seed sample (Q) turned dark within few minutes, whereas formazane precipitation was much less pronounced in water-germinated seedlings (Q_H_2_O) and virtually absent in vitamin B-germinated seedlings (Q_vit). To further examine this issue, NBT stainings were also conducted on fresh seedlings treated with water or vitamin B solution, respectively (directly after germination and washing). Formazane precipitation was clearly visible in water-treated but not in vitamin-treated samples, thus substantiating the observations made in dry material.

The data imply that (1) during germination the antioxidative machinery is induced, leading to efficient removal of superoxide. (2) Treatment with B vitamins appears to enhance this effect. (3) The different abundance of superoxide in Q_H_2_O (moderate levels), compared to Q_vit (barely visible) is maintained during industrial processing of seedlings to dry material.

These findings and interpretations are in agreement with previous reports. ROS, trapped in dry seeds, participate in the cracking of the seed shell. ROS are considered as being key actors in the regulation of germination and dormancy (Bailly et al. 2008b). As the seed germinates, the trapped superoxide is removed, thereby preventing oxidative damage. This removal appears to be particularly effective in the presence of one or several types of B vitamins. Likely candidates are vitamin B2, which is reportedly associated with redox balancing (Ashoori and Saedisomeolia 2014); but also vitamins B1, B6, and B9 known to have direct antioxidant activity in vitro (Gliszczynska-Swiglo 2006). In addition, vitamin B3 may indirectly be involved by providing NADH and NADPH to the antioxidant network.

### Antioxidant enzyme activities

The above results permit the assumption that certain antioxidant enzymes are differentially active in the three types of plant material.

**Figure 3:**
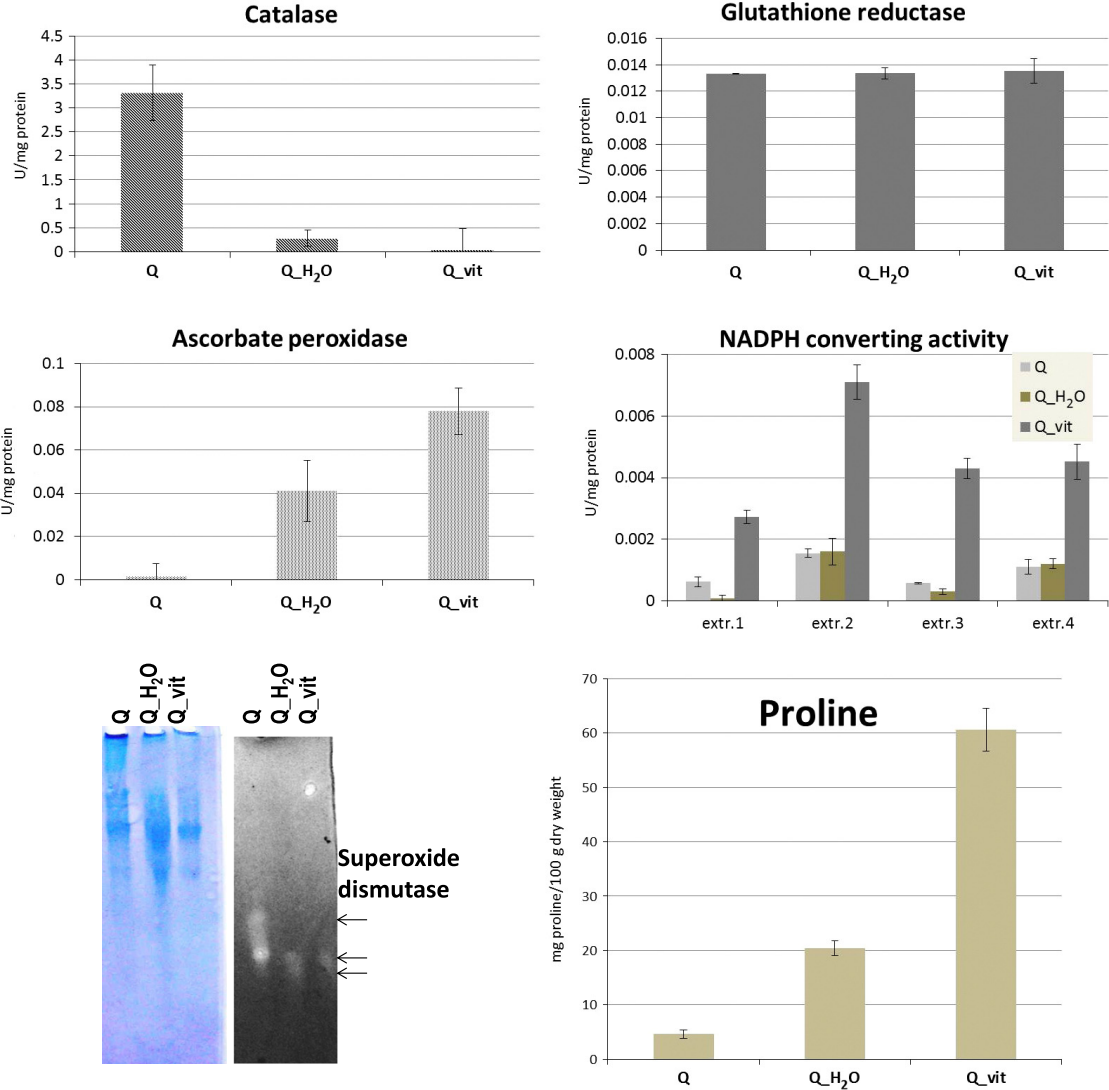
Antioxidant activities and proline content in quinoa seeds and seedlings. Antioxidant enzyme activities in quinoa seeds and seedlings. Experiments were repeated at least three times, and various protein extraction buffers were tested. One representative graph is shown for catalase, GR and APX. NADPH-converting activity is shown for four different protein extraction buffers. Data are shown as mean ± SD of three measurements that were conducted with independent protein extracts. For superoxide dismutase studies, duplicate sets of protein extracts were separated by native gel electrophoresis, followed by Coomassie staining (as loading control, left) or in-gel SOD activity assay (right). Three isoforms (arrows) can be seen. Proline quantification data are shown as mean ± SD of three independent measurements.

Activities of four major ROS-scavenging enzymes (superoxide dismutase; and catalase, glutathione reductase, ascorbate peroxidase) were therefore evaluated in protein extracts from plant material using in-gel activity assays and absorbance-based measurements, respectively (Fig.3).

### Superoxide dismutase

Superoxide dismutases convert toxic superoxide into molecular oxygen or hydrogen peroxide. Enzyme activities can be visualized on polyacrylamide gels. Because proteins are separated by nondenaturing gel electrophoresis, different SOD isoforms may be distinguished with this methodology. As shown in Figure3 (bottom, left), quinoa seed germination involves qualitative and quantitative changes in SOD activities. In agreement with a recent study in tobacco (Lee et al. 2013), a drop in SOD activity during seedling development can be noted. At least three SOD isoforms exist in seeds, which also show the strongest overall activity. The slow-migrating isoform is absent in extracts from seedlings. SOD profiles and activities are seemingly identical between Q_H_2_O and Q_vit (lane 2&3), a finding that was further corroborated by spectrophotometric measurements (data not shown).

The finding that low superoxide levels are not reflected by high SOD activities and vice versa points to major contribution by other factors of the antioxidant machinery.

### Catalase

Catalase dismutates H_2_O_2_ into water and oxygen.

Oxygen bubble formation was visible by eye in sample reactions containing seed protein extracts (Q). Accordingly, there was a progressive decrease in absorbance (240 nm). In contrast, catalase activity was barely detectable in protein extracts from seedlings.

Recently, a study in sunflower had correlated increased catalase activities with seed drying. The authors had noticed that, as the seed moisture content declines, catalase-encoding genes are activated, leading to the synthesis of enzymatically active proteins (Bailly et al. 2004). Our data point to a decrease in catalase activity during quinoa seed germination. Similar to sunflower (Bailly et al. 2004), seed moisture content, and catalase activity seem to be anticorrelated. The similarly low activities exhibited by both seedling samples (Q_ H_2_O, Q_vit) imply that catalase plays a minor role in ROS scavenging at this developmental stage, and that the enzyme remains unaffected by the vitamin treatment.

### Ascorbate peroxidase

Ascorbate conversion by ascorbate peroxidase (APX) was detectable in all samples. Compared to seeds, water-germinated seedlings displayed slightly higher APX activities. Interestingly, APX was markedly more active in vitamin B-treated seedlings, suggesting a particular contribution of the enzyme to ROS scavenging in this type of material.

Vitamin C (ascorbate), the substrate used by APX for peroxide detoxification, is likely present in sufficient amounts: As noted earlier, ascorbate levels increases during quinoa germination (Fuchs 1997). Elevated APX activities have been correlated with enhanced antioxidant capacities as well as with improved tolerance to drought, heat, salt and biotic stress (Sarowar et al. 2005; Gill and Tuteja 2010). One intriguing question is whether vitamin applications also provide a “health benefit” for plants. Stress exposure experiments are planned to reveal possible differences in plant fitness (Pitzschke, unpublished).

### Glutathione reductase

Glutathione is a crucial cellular antioxidant. It is used to detoxify hydrogen peroxide and in the process is oxidized to glutathione disulfide (GSSG). Glutathione reductase (GR), a flavin-dependent enzyme, restores the pool of glutathione by converting GSSG and NADPH to GSH and NADP+.

Enzymatic activities, evaluated by monitoring NADPH, were similar in Q, Q_H_2_O, and Q_vit. Assays with proteins isolated in alternative extraction buffers fully reproduced these results. GR activities in seeds are maintained during germination and apparently unaffected by the vitamin treatment, pointing to a stimulus-independent housekeeping function of the enzyme in redox balancing.

### NADPH-converting enzyme(s)

Interestingly, when GSSG was omitted from the reaction mix in GR assays, Q_vit protein extracts still exhibited substantial NADPH conversion, as revealed by the progressive decline in absorption at 340 nm (Fig.3). Irrespective of the protein extraction buffer used, this phenomenon was exclusively seen in reactions containing Q_vit extracts. Apparently, as a result of the vitamin treatment, activities of one or several NADPH-converting enzyme(s) other than glutathione reductase are induced. The nature of this enzyme, and thus the corresponding partner in the redox pair (NADPH + ?ox
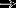
NADP + ?red) is elusive. Q_vit extracts apparently contain sufficient amounts of this unknown electron acceptor. NADPH oxidase, a membrane-bound enzyme that generates superoxide by transferring electrons form NADPH to molecular oxygen, is an unlikely explanation because similar activities were seen in extracts lacking or containing detergents. The fact that superoxide levels are reduced, not enhanced, in vitamin-treated seedlings also argues against NADPH oxidase. In order to systematically investigate the nature of these enzyme(s) and a possible involvement of the respiratory chain further studies employing specific inhibitors such as cyanide, salicylhydroxamic acid and diphenyleneiodonium are required. One likely candidate is monodehydroascorbate reductase, which uses NADPH to regenerate ascorbic acid for APX-mediated peroxide detoxification. Another possible explanation is NADPH conversion in the progress of proline biosynthesis. Given the fact that NADPH-dependent oxidoreductases employ nucleotide derivatives of vitamin B2 (riboflavin), FAD or FMN, as cofactor (Macheroux et al. 2011), our observations point to successful incorporation and derivation of the exogenously added vitamin. This in turn suggests that vitamin metabolites produced in plants may also be available as functional cofactors for human consumption. Clearly, medical evidence is needed to duly support this assumption.

One may argue that vitamins taken up during germination remain extracellular, that is, are not internalized inside the cells. However, standard media used for plant growth, plant cell culturing, and protoplast incubation (Murashige and Skoog 1962; Gamborg and Shyluk 1970) are supplemented with various B vitamins, implying that these molecules can pass/be transported through cell wall and plasma membrane.

### Proline

Among the proteinogenic amino acids, proline plays an exceptionally multi-faceted role in eukaryotic organisms. It is essential for primary metabolism, has structural functions and has been implicated in wound healing, antioxidative reactions and immune responses. Proline has therefore attracted special attention in medical and nutritional science (reviewed in Wu et al. 2011). Proline is also a research focus in plant physiology. Its accumulation has been reported during numerous abiotic and biotic stress conditions. Proline has osmoprotective activity, and stress-induced proline accumulation generally correlates with improved stress tolerance. Proline likely has direct ROS-scavenging activity (Smirnoff and Cumbes 1989), but it can also stabilize ROS scavenging enzymes (Hoque et al. 2008; Islam et al. 2009). Proline biosynthesis, a reductive pathway, involving the reduction in NADPH to NADP+, is important for maintaining appropriate levels of the electron acceptor NADP+ during stress. Plants with impaired proline biosynthesis and NADPH-NADP+ conversion are hypersensitive to stress (De Ronde et al. 2004).

Against this background we were encouraged to determine proline levels in quinoa seeds, water- and vitamin-treated seedlings. Dry plant material was freshly ground to a fine powder and assessed directly by the ninhydrin method (see methods). As shown in Figure3 (bottom, right), seeds contain approximately 5 mg proline per 100 g dry weight. In water-germinated seedlings, proline levels increased to app. 20 mg/100 g DW. Proline levels were elevated further in vitamin-treated seedlings (app. 60 mg/100 g DW). Consistent data were obtained with different batches of seeds and seedlings.

Proline has been implicated in the modulation of the intracellular redox environment and protection from oxidative stress in mammalian cells (Krishnan et al. 2008). The markedly higher proline levels in Q_vit as compared to seeds documents a further improvement of the nutritional value indirectly achieved by the vitamin treatment.

## Discussion and Concluding Remarks

This study provides a first insight into molecular changes during the early development of quinoa. The parallel comparative analysis of the three samples (Q, Q_H_2_O, Q_vit) allowed to distinguish between germination-related and vitamin B treatment-specific responses. Only effects caused by treatment with vitamin B complex solution were studied because material enriched in all eight B vitamins is most relevant for the food industry.

Young seedlings are very sensitive organisms. The fact that quinoa can develop in this artificial environment demonstrates its ability to achieving a balance between vitamin uptake, derivation, and degradation.

We propose the use of vitamin-treated plant tissue as next generation of food supplements. Flour of vitamin-treated seedlings can be industrially added to improve the nutritional value of food in a more natural manner. Young seedlings may therefore be considered promising “living factories” to convert synthetic vitamins into natural vitamin derivatives. However, direct evidence needs to be provided by metabolic profiling.

Quinoa responses to the vitamin treatment involve several metabolic adaptations. These adaptations (superoxide levels, enzyme activities) point to efficient prevention of oxidative damage and decay of biomolecules in vivo. An improvement in the nutritional value is furthermore documented by the enhanced proline content.

## Conflict of Interest

None declared.
